# Sequential combination with ropeginterferon alfa-2b and anti-PD-1 treatment as adjuvant therapy in HBV-related HCC: a phase 1 dose escalation trial

**DOI:** 10.1007/s12072-025-10824-4

**Published:** 2025-04-05

**Authors:** Albert Qin, Ming-Chih Ho, Chan-Yen Tsai, Chun-Jen Liu, Pei-Jer Chen

**Affiliations:** 1 Medical Research and Clinical Operations, PharmaEssentia Corporation, Taipei, Taiwan; 2 Department of Surgery, National Taiwan University Hospital, Taipei, Taiwan; 3 Department of Surgery, National Taiwan University Hospital Hsin-Chu Branch, Hsinchu, Taiwan; 4 Department of Internal Medicine, Graduate Institute of Clinical Medicine, National Taiwan University College of Medicine, No. 7, Chung Shan South Rd., Taipei, Taiwan; 5 Hepatitis Research Center, National Taiwan University Hospital, Taipei, Taiwan

**Keywords:** Hepatocellular carcinoma (HCC), Hepatitis B virus (HBV), Anti-PD-1, Interferon, Ropeginterferon alfa-2b (ropeg), Nivolumab, Adjuvant therapy, Cancer recurrence, Alanine transaminase (ALT) flares, Clinical trial

## Abstract

**Background/purpose:**

Post-operative recurrence is a major clinical challenge with hepatocellular carcinoma (HCC). While currently unapproved, anti-programmed cell death 1 (PD-1) and anti-vascular endothelial growth factor combination adjuvant therapy showed promise. We initiated a phase I trial using sequential treatment with ropeginterferon alfa-2b (ropeg), a novel interferon-based antiviral and antitumor agent, followed by anti-PD-1 therapeutic antibody nivolumab as an adjuvant therapy for hepatitis B virus (HBV)-related HCC.

**Methods:**

Patients who underwent surgical resection of HBV-related HCC with curative intent received sequential therapy with six doses of ropeg every two weeks at 450 μg, followed by three doses of nivolumab escalating from 0.3 to 0.75 mg/kg every two weeks. Safety, HBV surface antigen (HBsAg) loss or decrease, anti-HBV surface (HBs) antibodies, cancer recurrence, and survival were evaluated.

**Results:**

Fifteen eligible patients were enrolled. Most adverse events (AEs) were mild or moderate and no severe or serious AEs were observed. Alanine transaminase flares, including one grade 3 event as dose-limiting toxicity, were noted in five cases and the final recommended dose for anti-PD1 was determined at 0.75 mg/kg. Interestingly, all five cases had HBsAg clearance or reduction. All patients in the study were alive without cancer recurrence during a median follow-up of 1024 days with six patients surviving > 4 years and three for > 5 years.

**Conclusions:**

This phase I trial supports the safety and clinical efficacy of sequential treatment with ropeg and nivolumab in post-resection HBV-related HCC. This regimen holds promise for further adjuvant therapy trials in HCC, both HBV-related and other types.

**Graphic abstract:**

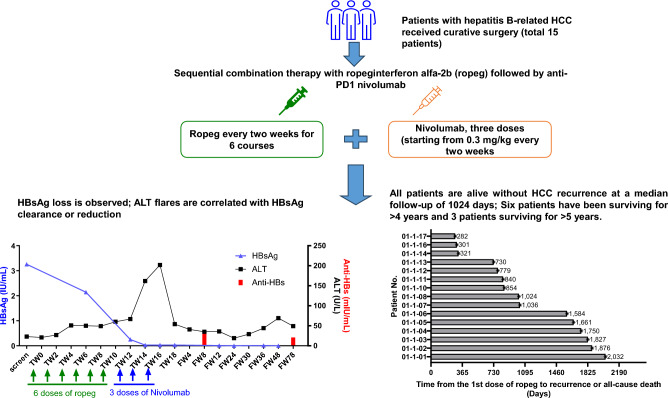

**Supplementary Information:**

The online version contains supplementary material available at 10.1007/s12072-025-10824-4.

## Introduction

Hepatocellular carcinoma (HCC) is a deadly cancer worldwide [1]. Most HCC cases are associated with well-known risk factors, including chronic viral hepatitis or non-viral metabolic or alcoholic hepatitis. Chronic hepatitis B virus (HBV) infection is the leading cause of HCC worldwide, followed by chronic infection with hepatitis C virus (HCV) [2]. Treatment options for HCC depend upon disease stage. For early-stage HCC where cure is the goal, surgical resection is the primary option with local ablation or liver transplantation used in some cases [3]. Unfortunately, HCC recurrence rates after liver surgical resection are notably high [4]; approximately 50% recur at three years and 70% at 5 years post-resection, respectively [5]. The time to recurrence shows a bimodal distribution of early and late recurrences [6]. Early recurrence accounts for more than 70% of post-surgical recurrence and typically occurs within two years of surgery [6, 7]. Early recurrence is generally believed to be due to residual tumor cells giving rise to new intrahepatic metastasis, whereas late recurrence (typically occurring 4–5 years post-surgery) is believed to result from de novo lesions [6, 7]. For patients with HBV-related HCC, the risk of recurrence is even greater as HBV DNA integration can potentiate cancer recurrence with oncogene activation [8]. Currently, no approved adjuvant therapies are available to control recurrence despite recent progress using combination therapy with anti-programmed cell death 1 (PD-1) and anti-vascular endothelial growth factor (VEGF) antibodies for 12 months. This combination therapy has shown a promising reduction of one-year HCC recurrence and long-term follow-up is ongoing for determining the impact on overall survival [9].

Interferons (IFNs), including type 1 IFNs alpha (IFN-α) and beta (IFN-β), are anticancer agents and IFN-α preparations have been used as adjuvant therapy for patients after hepatic resection of HCC and found to be safe. However, traditional IFN therapy has been shown to have limited clinical efficacy [10, 11] which may be due to several reasons including subtherapeutic IFN levels due to limited tolerability, or the need for additive/synergistic therapies to enhance adaptive immunity. Immuno-oncology combination therapies are currently being intensively explored in this clinical setting [12]. Immune checkpoint inhibition using anti-PD-1 antibodies is approved for the treatment of patients with advanced HCC [13]. Theoretically, PD-1 blockade could reactivate exhausted T cell immunity and suppress viral oncogene-induced tumor formation [12, 14, 15]. Ropeginterferon alfa-2b (ropeg) is a new polyethylene glycol-conjugated (PEGylated) IFN-based agent with anti-viral and anti-tumor activities and is tolerable at high doses in patients [16–18]. We therefore explored the potential of a sequential combination therapy approach using ropeg at a high dose level of 450 μg followed by an anti-PD-1 therapeutic antibody as an adjuvant therapy in HBV-related HCC [19]. Either IFN or anti-PD1 treatment alone can occasionally induce clinically significant hepatitis in patients with HBV [14, 20]. To minimize toxicities and maximize efficacy, we explored using a fixed dose of ropeg treatment at 450 μg followed by a dose escalation of the anti-PD-1 antibody nivolumab starting from a low dose of 0.3 mg/kg. Animal modeling data and preliminary phase 1 clinical data suggested that a sequential ropeg treatment once every two weeks for 6 courses followed by nivolumab every two weeks for 3 courses is safe and potentially effective in reinvigorating innate and adaptive immunities [19]. Here we report the phase 1 study results, including the detailed baseline demographic information of patients in each dose escalation cohort, together with safety profiles (especially hepatitis flares), anti-HBV activities including hepatitis B surface antigen (HBsAg) loss or decrease, and long-term survival data.

## Material and methods

### Patients

Patients received surgical resection for HCC within eight weeks prior to screening. Eligible patients must have positive HBsAg with undetectable HBV DNA (HBV Roche Cobas TaqMan test) in the presence or absence of nucleos[t]ide analogs (NA)-based antiviral therapy, compensated liver disease, normal fundoscopic examination, and Eastern Cooperative Oncology Group Performance Status score of 0 to 1. Major exclusion criteria included HCC unrelated to HBV, vascular invasion of HCC on imaging diagnosis, a concurrent active malignancy other than HCC, and a history of transcatheter arterial embolization or chemoembolization, transcatheter arterial infusion, or chemolipiodolization in combination with surgery.

### Study design and treatments

**Fig. 1:**
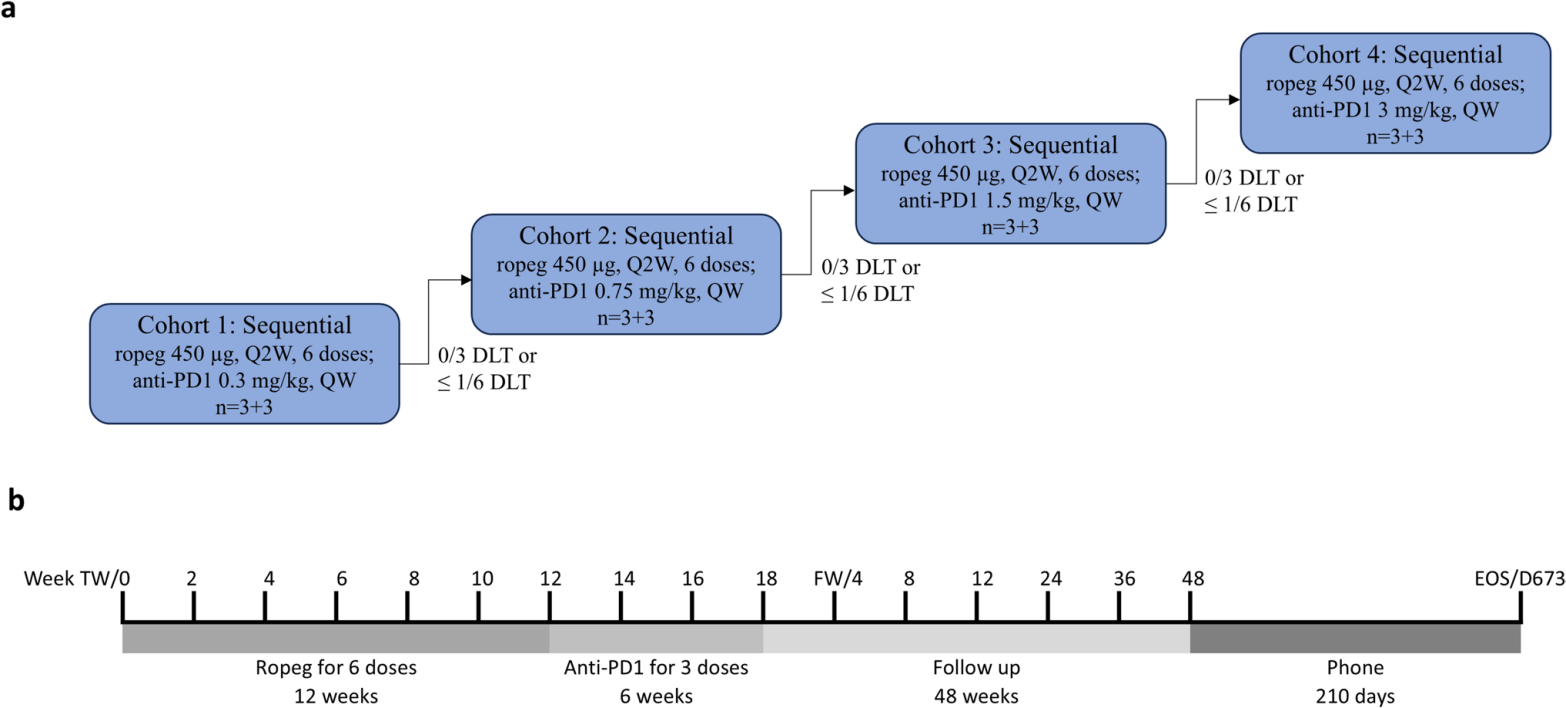
Dose escalation schema (**a**) and time scale (**b**) of the phase I study

This phase 1 study aimed to evaluate the safety and tolerability and define the maximum tolerated dose (MTD) and recommended phase 2 dosing of the sequential administration of ropeg and nivolumab in patients who had received curative intent surgery for HBV-related HCC. The study was conducted at the National Taiwan University Hospital in Taiwan (approval number: 201710061MIPB) and is registered in ClinicalTrials.gov (NCT04233840). The detailed study design was previously reported [19]. Eligible patients were enrolled into a three-case cohort to receive six doses of ropeg subcutaneously at 450 μg once every two weeks, followed by three doses of nivolumab once every two weeks at 0.3 mg/kg for Cohort 1 and planned standard 3 + 3 dose escalation criteria [21] to 0.75 mg/kg (Cohort 2), 1.5 mg/kg (Cohort 3), and 3 mg/kg (Cohort 4) until the MTD is reached (Fig. 1). Nivolumab was administrated via intravenous infusion over 60 min. The trial was initiated in 2019, but recruitment was delayed during the COVID-19 pandemic.

### Assessments

Adverse events (AEs) were continuously monitored during the treatment period and for up to 48 weeks after the completion of study treatment. All AEs were coded using preferred terms according to the Medical Dictionary for Regulatory Activity terminology. AE severity was graded using the National Cancer Institute Common Terminology Criteria for Adverse Events version 4.03.

Disease progression was continuously monitored using computed tomography (CT) or magnetic resonance imaging (MRI) every 12 to 30 weeks after the completion of the study treatment. Overall survival and recurrence-free survival (defined as recurrence or death from any cause) were continuously assessed.

### Statistical analysis

General statistical summaries were used. For categorical variables, frequencies and percentage were presented. For continuous variables, number of patients, mean, standard deviation (SD), median, minimum, and maximum were presented if appropriate. For endpoints with measurements over time, the statistics were also presented by timepoint.

For recurrence-free survival, the Kaplan–Meier method was used to estimate the accumulative distribution. The starting point for time-to-event analyses was at the first dose of ropeg.

## Results

### Demographics and baseline characteristics

A total of 15 eligible patients were enrolled. The mean patient age was 61.8 (standard deviation [SD]: ± 10.8) years (Table 1). Seven (47%) patients had liver cirrhosis. All patients had one or two tumor nodules prior to surgical resection with a median maximum tumor size of 3.8 cm. Twelve patients were categorized as having TNM stage I/II HCC, one was stage III, and the other two were stage IV. For the patients with stage IV tumors, one had a peritoneal metastasis and the other had an abdominal wall metastasis. According to Barcelona clinic liver cancer classification (BCLC), thirteen patients were classified as stage A and two were stage B. No patients had tumors with vascular invasion, and none had received prior neoadjuvant therapy for HCC. Two patients had a preoperative alpha-fetoprotein (AFP) level ≥ 200 ng/ml. Mean pre-operative aspartate transaminase (AST) and alanine transaminase (ALT) levels were 31.1 (SD: ± 15.6) U/l and 33.0 (SD: ± 23.1) U/l, respectively. All local and metastasized tumors were completely removed in the surgical excision, with no residual tumor remaining in the post-operative tumor assessment by CT/MRI and AFP at two months. After operation, the mean (SD) AFP reduction was -133.32 (338.61) ng/ml (Table 1 and supplemental Fig. 1a). For those with baseline AFP ≥ 200 ng/ml, the mean reduction was − 965.09 (84.39) ng/ml (*p* < 0.05, compared to baseline level, supplemental Fig. 1b). The post-operative AST and ALT levels were 5.3 (SD: ± 5.6) U/l and 29.3 (SD: ± 22.7) U/l, respectively. All patients had undetectable HBV DNA, and 14/15 (93%) patients had received NA therapies prior to screening. After enrollment, anti-HBV NA therapies were given to all patients. The mean pre- and post-operative HBsAg levels were 3644.0 (SD: ± 2243.7) IU/ml and 1243.0 (SD: ± 2212.8) IU/ml, respectively.

**Table 1:** Patient demographics and baseline characteristics

Demographics/baseline characteristics	Ropeg + Anti-PD1 *N* = 15		
	Cohort 1 *N* = 6	Cohort 2 *N* = 9	Total *N* = 15
Demographics			
Age, years			
Mean (SD)	64.2 (6.6)	59.5 (14.1)	61.8 (10.8)
Range (min–max)	53–72	40–75	40–75
Gender			
Male, *n* (%)	5 (83%)	6 (67%)	11 (73%)
Female, *n* (%)	1 (17%)	3 (33%)	4 (27%)
Liver cirrhosis			
Yes	3 (50%)	4 (44%)	7 (47%)
No	3 (50%)	5 (56%)	8 (53%)
HCC status prior to surgical operation			
Median time from the 1st HCC diagnosis to surgical operation	– 4 (8437, – 12)	– 1 (2570, – 7)	– 3 (8437, – 12)
TNM stage			
I	4 (67%)	6 (67%)	10 (67%)
II	2 (33%)	0 (0%)	2 (13%)
III	0 (0%)	1* (11%)	1* (7%)
IV	0 (0%)	2** (22%)	2** (13%)
BCLC stage			
A	5 (84%)	8 (89%)	13 (87%)
B	1 (16%)	1 (11%)	2 (13%)
C	0 (0%)	0 (0%)	0 (0%)
D	0 (0%)	0 (0%)	0 (0%)
Tumor nodule			
Single	5 (83%)	8 (89%)	13 (87%)
Two	1 (17%)	1 (11%)	2 (13%)
Maximum tumor size, cm, median (max, minimum)	3.9 (10.5, 1.7)	3.0 (8.3, 2.0)	3.8 (10.5, 1.7)
≥ 5 cm	2 (33%)	2 (22%)	4 (27%)
< 5 cm	4 (67%)	7 (78%)	11 (73%)
Vascular invasion			
Yes	0 (0%)	0 (0%)	0 (0%)
No	6 (100%)	9 (100%)	15 (100%)
Extent of liver resection			
1 segment	5 (83%)	8 (89%)	13 (87%)
2 segment	1 (17%)	1 (11%)	2 (13%)
Adjuvant therapy for HCC			
Yes	6 (100%)	9 (100%)	15 (100%)
No	0 (0%)	0 (0%)	0 (0%)
AFP level, ng/ml			
Mean (SD)	492.1 (561.3)	13.6 (16.0)	173.1 (376.3)
Median (max, min)	467.2 (1031.0, 3.1)	5.8 (43.1, 2.5)	9.0 (1031.0, 2.5)
≥ 200 ng/ml	2 (33%)	0 (0%)	2 (13%)
< 200 ng/ml	4 (67%)	9 (100%)	13 (87%)
AST level prior to the operation, U/L			
Mean (SD)	33.0 (18.2)	29.8 (14.6)	31.1 (15.6)
Median (max, min)	25.0 (64.0, 19.0)	24.0 (67.0, 20.0)	25.0 (67.0, 19.0)
ALT level prior to the operation, U/L			
Mean (SD)	31.2 (17.9)	34.1 (27.1)	33.0 (23.1)
Median (max, min)	28.5 (63.0, 13.0)	27.0 (104.0, 12.0)	27.0 (104.0, 12.0)
HBsAg level prior to the operation, IU/mL			
Mean (SD)	4537.8 (2159.7)	3048.2 (2212.7)	3644.0 (2243.7)
Median (max, min)	5457.9 (5849.2, 224.1)	2386.4 (6630.4, 119.9)	4560.0 (6630.4, 119.9)
Baseline characteristics at the 1st dose of ropeg			
Time from surgical operation to the post-operation tumor assessment^#^, days, median (max, minimum)	35.5 (65.0, 22.0)	39.0 (52.0, 26.0)	38.0 (65.0, 22.0)
Number of residual tumor nodules after surgical operation	0 (0%)	0 (0%)	0 (0%)
Time from the surgical operation to the 1st dose of ropeg, days, median (max, minimum)	60.50 (79.0, 51.0)	64.0 (78.0, 42.0)	62.0 (79.0, 42.0)
AFP level, ng/ml			
Mean (SD)	6.9 (6.5)	4.2 (5.0)	5.3 (5.6)
Median (max, min)	4.3 (17.0, 1.0)	2.4 (17.2, 1.0)	2.5 (17.2, 1.0)
≥ 200 ng/ml	0 (0%)	0 (0%)	0 (0%)
< 200 ng/ml	6 (100%)	9 (100%)	15 (100%)
Absolute change of AFP level from pre-operation to post-operation			
Mean (SD)	– 321.33 (500.08)	– 7.98 (12.72)	– 133.32 (338.61)
Median (max, min)	0.04 (1.13, – 1024.76)	– 1.30 (1.09, – 33.41)	– 0.60 (1.13, – 1024.76)
AST level prior to the 1st doe of ropeg, U/L			
Mean (SD)	28.3 (15.9)	4.2 (5.0)	5.3 (5.6)
Median (max, min)	23.0 (60.0, 18.0)	2.4 (17.2, 1.0)	2.5 (17.2, 1.0)
ALT level prior to the 1st doe of ropeg, U/L			
Mean (SD)	29.0 (27.4)	29.4 (20.8)	29.3 (22.7)
Median (max, min)	17.5 (84.0, 13.0)	23.0 (81.0, 12.0)	21.0 (84.0, 12.0)
HBsAg level prior to the 1st dose of ropeg, IU/mL			
Mean (SD)	962.7 (1601.9)	1429.8 (2620.5)	1243.0 (2212.8)
Median (max, min)	236.4 (4166.7, 53.6)	168.3 (8057.8, 3.3)	232.4 (8057.8, 3.3)
Receiving NA-based antiviral therapy (%)	6 (100%)	9 (100%)	15 (100%)

Note: BCLC: Barcelona clinic liver cancer classification; HCC: hepatocellular carcinoma; TNM: tumor, node, metastasis; ropeg: ropeginterferon alfa-2b; AFP: alpha-fetoprotein; AST: aspartate transaminase; ALT: alanine transaminase; HBsAg: hepatitis B surface antigen; NA: nucleos[t]ide analogs

*TNM stage of this patient was T3N0M0

**TNM stages of these two patients were T4N0M1 and T1aN1M1. One patient had a tumor metastasized to peritoneum and one had abdominal wall metastasis

^#^The methods of tumor assessment included computed tomography (CT) and magnetic resonance imaging (MRI)

### Cohort study for anti-PD1 dose escalation: establishment of the recommended phase 2 dosing regimen

**Fig. 2:**
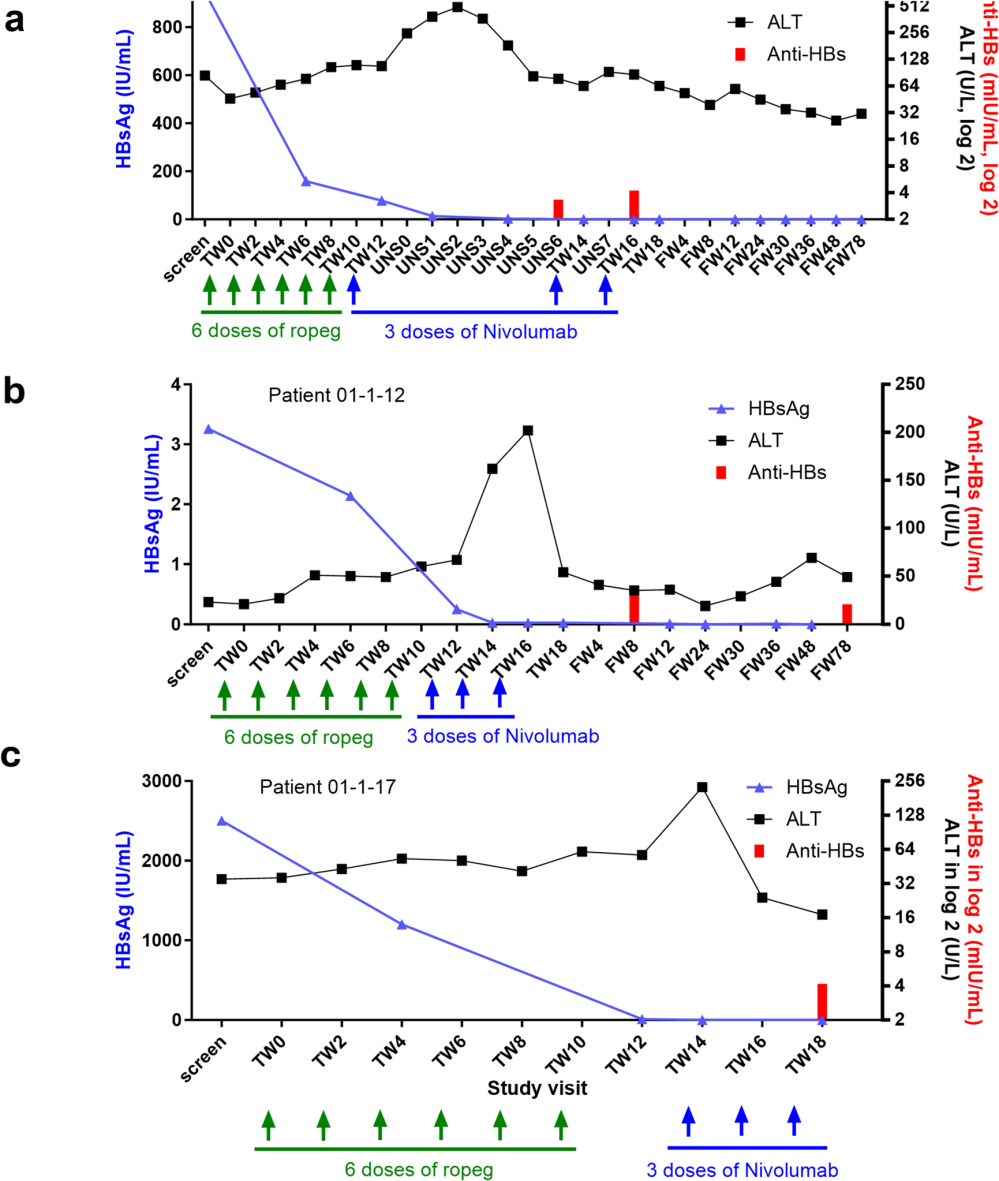
HBsAg, ALT, and anti-HBs antibody levels overtime in individual patient who achieved HBsAg clearance during the study period (**a**–**c**). TW: treatment week; UNS: unscheduled site visit; FW: follow-up week; HBsAg: hepatitis B surface antigen; ALT: alanine transaminase; Anti-HBs: hepatitis B surface antibody

**Fig. 3:**
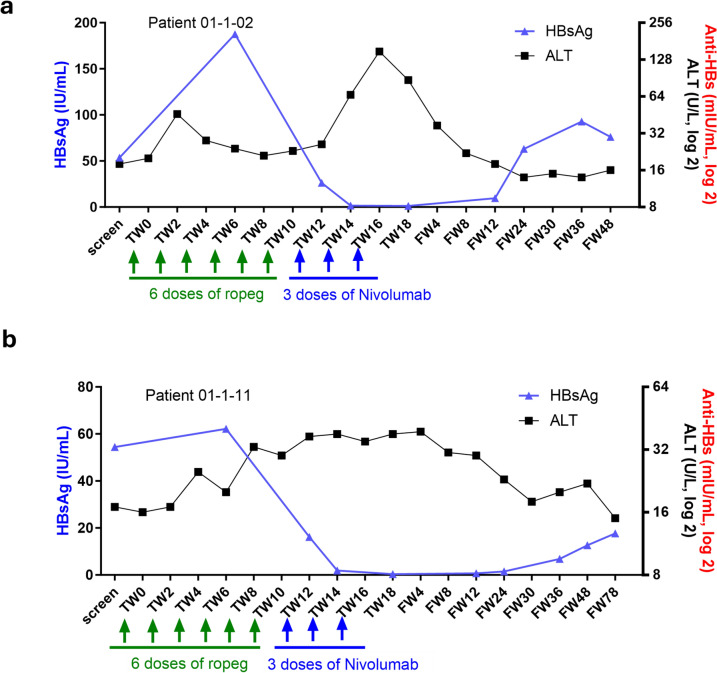
HBsAg, ALT, and anti-HBs antibody levels overtime in individual patient who had a two-log HBsAg reduction but no clearance during the study period (**a**, **b**). TW: treatment week; FW: follow-up week; HBsAg: hepatitis B surface antigen; ALT: alanine transaminase; Anti-HBs: hepatitis B surface antibody

In Cohort 1, three eligible patients were first enrolled to receive six doses of ropeg at 450 μg once every two weeks followed by three doses of nivolumab at 0.3 mg/kg. The most common AEs were ALT increase, AST increase, and pyrexia at mild to moderate grade (Table 2). Two patients experienced grade 1/2 ALT elevations which occurred during the switch from ropeg to nivolumab treatment, and interestingly, one of these patients also showed a synchronous two-log reduction in HBsAg level (Fig. 3a). In addition, another patient who experienced a dose-limiting toxicity (DLT) as a grade 3 ALT elevation after the first dose of nivolumab, experienced subsequent HBsAg loss and the induction of hepatitis B surface antibody (anti-HBs antibody) (Fig. 2a). Due to the occurrence of this DLT, three additional patients were enrolled to receive sequential treatment with ropeg at 450 μg for six doses followed by nivolumab at 0.3 mg/kg for 3 doses in Cohort 1. For the three additional patients, all AEs were mild to moderate and no DLT occurred (Table 2). The safety profile of the sequential regimen of ropeg at 450 μg followed by nivolumab at 0.3 mg/Kg was deemed acceptable.

**Table 2:** Summary of adverse events (AEs) in the study

AEs, *n* (%)	Cohort 1 (*N* = 6)*				Cohort 2 (*N* = 9)^#^						Total (*N* = 15)
	Patients 01-1-01, 01-1-02, 01-1-03		Patients 01-1-04, 01-1-05, 01-1-06		Patients 01-1-07, 01-1-08, 01-1-10		Patients 01-1-11, 01-1-12, 01-1-13		Patients 01-1-14, 01-1-16, 01-1-17		
Any AE	3 (100%)		3 (100%)		3 (100%)		3 (100%)		3 (100%)		15 (100.0%)
Any Grade3 AE	2 (66.7%)		0 (0%)		2 (66.7%)		0 (0%)		2 (66.7%)		6 (40.0%)
Any SAE	0 (0%)		0 (0%)		0 (0%)		0 (0%)		0 (0%)		0 (0%)

AEs occurring in > 10% of patients *n* (%)	Grade 1/2	Grade 3	Grade 1/2	Grade 3	Grade 1/2	Grade 3	Grade 1/2	Grade 3	Grade 1/2	Grade 3	
ALT increased	2 (66.7%)	1 (33.3%)^&^	0 (0%)	0 (0%)	0 (0%)	0 (0%)	2 (66.7%)	0 (0%)	2 (66.7%)	1 (33.3%)	8 (53.3%)
AST increased	2 (66.7%)	1 (33.3%)^&^	0 (0%)	0 (0%)	0 (0%)	0 (0%)	2 (66.7%)	0 (0%)	2 (66.7%)	1 (33.3%)	8 (53.3%)
Pyrexia	2 (66.7%)	0 (0%)	2 (66.7%)	0 (0%)	0 (0%)	0 (0%)	2 (66.7%)	0 (0%)	1 (33.3%)	0 (0%)	7 (46.7%)
Fatigue	1 (33.3%)	0 (0%)	1 (33.3%)	0 (0%)	0 (0%)	0 (0%)	2 (66.7%)	0 (0%)	1 (33.3%)	0 (0%)	5 (33.3%)
Neutrophil count decreased	0 (0%)	1 (33.3%)	1 (33.3%)	0 (0%)	0 (0%)	0 (0%)	1 (33.3%)	0 (0%)	1 (33.3%)	2 (66.7%)	5 (33.3%)
Anorexia	1 (33.3%)	0 (0%)	0 (0%)	0 (0%)	0 (0%)	1 (33.3%)^&^	0 (0%)	0 (0%)	0 (0%)	0 (0%)	2 (13.3%)
Insomnia	1 (33.3%)	0 (0%)	0 (0%)	0 (0%)	0 (0%)	0 (0%)	1 (33.3%)	0 (0%)	0 (0%)	0 (0%)	2 (13.3%)
Pruritus	0 (0%)	0 (0%)	1 (33.3%)	0 (0%)	0 (0%)	0 (0%)	0 (0%)	0 (0%)	1 (33.3%)	0 (0%)	2 (13.35)

Note: % = percentage of patients with *N* as the denominator

Patients in Cohort 1 received six doses of ropeg at 450 μg once every two weeks followed by three doses of nivolumab at 0.3 mg/kg. Patients in Cohort 2 received six doses of ropeg at 450 μg once every two weeks followed by three doses of nivolumab at 0.75 mg/kg

AE: adverse event; SAE: serious adverse event

*Patients 01-1-01, 01-1-02, and 01-1-03 were initially enrolled in Cohort 1. Because of the occurrence of one DLT in the first three patients, three additional patients, i.e., Patients 01-1-04, 01-1-05, and 01-1-06, were enrolled in Cohort 1 based on the protocol pre-specified 3 + 3 dose escalation criteria

^#^Patients 01-1-07, 01-1-08, and 01-1-10 were initially enrolled in Cohort 2. Because of the occurrence of one DLT in the first three patients, Patients 01-1-11 to 01-1-17 were enrolled in Cohort 2 based on the protocol pre-specified 3 + 3 dose escalation criteria

^&^The occurrences of Grade 3 ALT and Grade 3 AST increase in Patient 01-1-03 and Grade 3 anorexia in Patient 01-1-10 were identified as DLTs

The study therefore proceeded to Cohort 2 with nivolumab dose escalating from 0.3 mg/kg to 0.75 mg/kg. The first three patients in Cohort 2 had mild to moderate AEs including weight loss (33.3%), oral ulcer (33.3%), palmar-plantar erythrodysesthesia syndrome (33.3%), and facial nerve disorder (33.3%). Grade 3 anorexia lasting for more than seven days was identified as a DLT for ropeg in one patient. Therefore, three additional patients were enrolled in Cohort 2. Overall, the sequential regimen with nivolumab at 0.75 mg/kg was well-tolerated. However, there were grade 1/2 ALT and AST elevations in two patients. One of them also experienced HBsAg clearance (Fig. 2b). Therefore, six doses of ropeg at 450 μg followed by three doses of nivolumab at 0.75 mg/kg as used in Cohort 2 were determined to be the MTD for the adjuvant sequential therapy [19]. Three more patients were further treated at the MTD. HBsAg loss coincident with Grade 3 ALT and AST elevations was observed in one of the three patients. The MTD was thus established as the recommended phase 2 dosing regimen.

All patients experienced at least one AE after treatment. Most AEs were mild or moderate (Table 2). No Grade 4 or 5 AEs or serious AEs were observed. Six patients (40.0%) experienced Grade 3 AEs; the most frequent being ALT increase (53.3%) and AST increase (53.3%), followed by pyrexia (46.7%), fatigue (33.3%), and neutrophil count decrease (33.3%).

### HBsAg clearance was associated with ALT flares

Acute elevation of ALT, i.e., ALT flare, may be a manifestation of reinvigorating host immunity in clearing the HBV-infected hepatocytes [22–24]. HBsAg loss, ALT levels, and anti-HBs antibodies were therefore carefully assessed in this study. Interestingly, the HBsAg levels started to decline after ropeg therapy (Fig. 2). When switching to anti-PD-1 treatment, there was a rapid ALT surge accompanied with a rapid HBsAg reduction. As noted, the ALT flare was notable in all three patients who had HBsAg clearance, or HBsAg loss (Fig. 2). Induction of anti-HBs antibody was observed in the three patients after the occurrence of ALT flare. Furthermore, two patients who had an approximate two-log reduction in the HBsAg levels also had ALT flares. In these patients, the HBsAg levels rebounded after completion of nivolumab treatment (Fig. 3).

### Long-term follow-up for HBV-related HCC: recurrence and patient survival

**Fig. 4:**
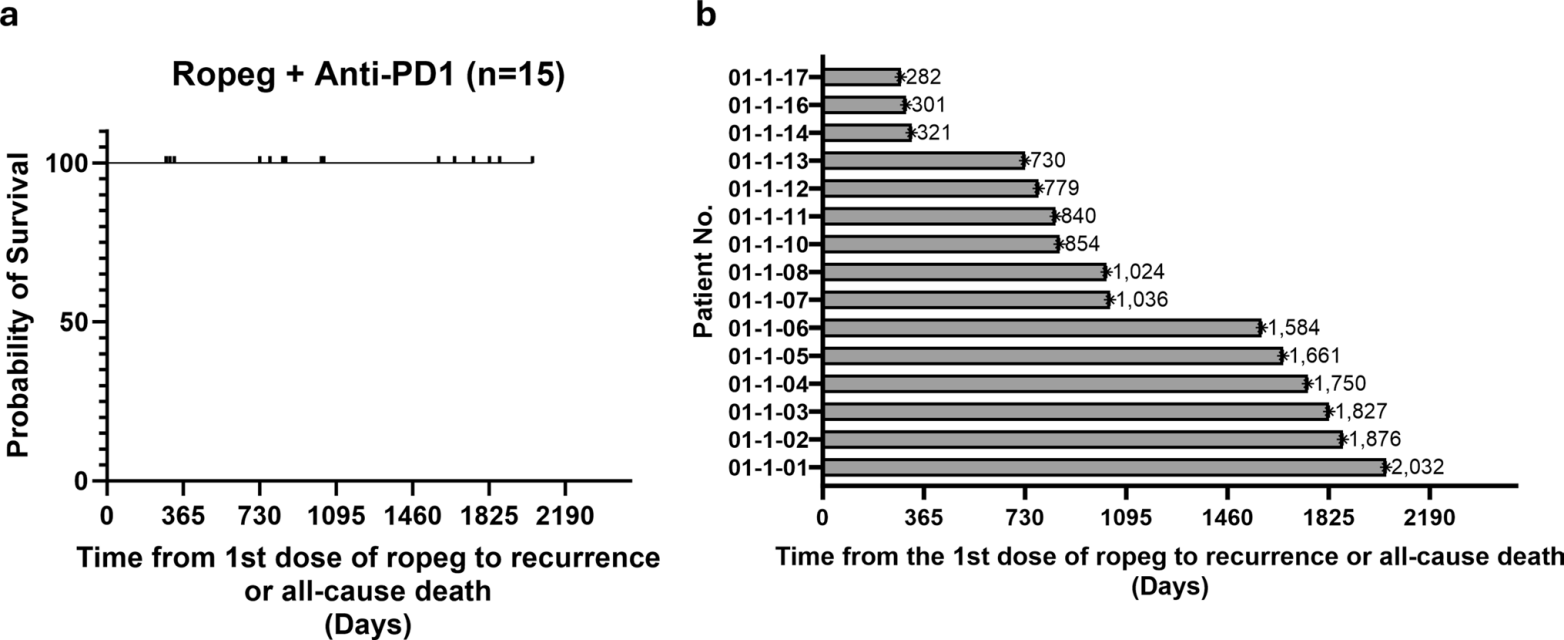
Survival status of hepatocellular carcinoma (HCC) patients receiving sequential therapy with ropeg and anti-PD-1 antibody for inhibiting recurrence of HCC. **a** Recurrence-free survival curve of all 15 patients. **b** Recurrence-free survival of individual patient. The symbols of ^▐^ and * represent the censor of event in (**a** and **b**), respectively

All patients were alive without HCC recurrence at a median follow-up of 1024 days (range 282 to 2032 days, Fig. 4a). The median overall survival and median recurrence-free survival have not been reached. Among the 15 patients enrolled, twelve (80%) have survived longer than two years without HCC recurrence (Fig. 4b), including six patients surviving more than four years and three patients surviving more than 5 years without any recurrence.

## Discussion

HCC is a common cause of cancer-related death. Most HCC cases are associated with chronic viral hepatitis with HBV infection as the leading risk factor [1]. Surgical resection remains the standard of care for early-stage HCC, but post-surgical recurrence rates are high [4, 5]. Approximately 70% of the cases exhibit early occurrence, i.e., usually within two years following surgery [6, 7]. Residual tumor cells are believed to be the cause of the early recurrence. Late recurrence which typically occurs at 4–5 years after the surgical resection possibly results from de novo lesions [6, 7]. Residual HBV infection in HBV-related HCC is a risk factor for post-surgical cancer recurrence via multiple ways including the activation of oncogenes [8]. Therefore, a high unmet medical need exists for novel and effective adjuvant regimens to suppress the post-surgical recurrence of HBV-related HCC. Our completed phase 1 study and long-term follow-up of the sequential combination use of ropeg at a tolerable, high dose of 450 μg followed by anti-PD-1 antibody nivolumab at low doses demonstrate the utility of this regimen as adjuvant therapy following hepatic resection of HBV-related HCC. This adjuvant regimen also induced notable anti-HBV activities, potentially clearing residual HBV infection as indicated by HBsAg loss or decrease associated with ALT flares following surgery in HBV-related HCC. At a median follow-up period of 1024 days, all 15 patients were alive without HCC recurrence following adjuvant sequential combination therapy. Six of these patients have survived more than four years without HCC recurrence and three have survived more than three years. These results further suggest that this sequential adjuvant combination regimen could inhibit both early recurrence as well as the late recurrence in HBV-related HCC.

Immune checkpoint inhibition with anti-PD-1 or anti-PD-L1 therapeutic antibodies is approved for the treatment of patients with advanced HCC [13]. While these antibodies could also be potentially used as adjuvant therapy to inhibit HCC recurrence, toxicities and insufficient potency may limit their use as a single agent in this setting. In patients with HBV-related HCC, anti-PD-1 monotherapy at the approved dosing regimen induces clinically significant hepatotoxicity [14]. Recently, the combination of anti-PD-1 antibody atezolizumab and anti-VEGF antibody bevacizumab was shown to improve recurrence-free survival in patients with resected or ablated high-risk HCC in a phase 3 trial [9]. With a median follow-up of 17.4 months (approximately 522 days), HCC recurrence was seen in 40% of patients in the control group vs 33% in the combination treatment group [9]. While this result was encouraging, a longer-term follow-up is required. Our phase 1 trial showed promising outcomes with a longer median follow-up of 1024 days (34.1 months).

Ropeg is a novel PEGylated IFN-α-based therapy approved for the treatment of a myeloproliferative neoplasm, polycythemia vera, and can potentially activate a network of tumor suppressors or their related proteins by binding to IFN receptors on tumor cells to inhibit the neoplastic phenotype [16]. IFN-α and IFN-β are involved in the cell cycle-based anti-cancer surveillance and can bind to their receptors on tumor cells to activate a checkpoint to inhibit the cell cycle progression accompanied by senescence and suppressed tumorigenicity [25, 26]. IFNs can also bind to their receptors on immune cells in the immune system to activate the natural killer cell-dependent and CD8^+^ T-cell-mediated anti-tumor responses [27, 28]. Additionally, IFN-α can modify the tumor microenvironment and recruit cytotoxic CD8^+^ T-cells to potentiate anti-PD-1-induced immune responses [29, 30]. In the current study, we used ropeg at a high dose of 450 μg, which was well-tolerated and active in our previous phase II trials in patients with chronic viral hepatitis including hepatitis B [18, 31]. Ropeg at this dose level can potentially elicit direct or indict anti-tumor activities including potentiating anti-PD-1 antibody-induced antitumor, immunologic response as discussed above.

Several risk factors have been shown to be correlated with recurrence, including vascular invasion, serum AFP level, tumor, size, and multiple lesions [6, 7]. In this study, six patients were identified as being high-risk for recurrence based on baseline tumor size ≥ 5 cm, multiple tumor lesions, metastasis, or baseline AFP ≥ 200 ng/ml. Of the 6 high-risk patients, four achieved a recurrence-free survival of longer than four years. This result suggests that the sequential regimen of ropeg and nivolumab is effective at suppressing post-operative recurrence in high-risk patients with HBV-related HCC.

Compared to anti-HBV NA therapies, IFN-based treatment can lead to higher rates of HBsAg seroconversion and reduce the risk for the transformation of HCC at a greater level in patients with chronic HBV infection [32]. We found that mean HBsAg levels decreased over time with the combination regimen of ropeg and nivolumab in animal models and in patients with post-resection HBV-related HCC [19]. In this study, we additionally found an association between ALT flares occurring just before the HBsAg loss or reduction. Interestingly, anti-HBs antibodies were also induced after the ALT flares. Our results are consistent with the data reported by others [22, 23], suggesting that ALT flares can be a mechanistic indicator of reviving host immunity against chronic hepatitis B. As HBV is a non-cytopathic virus, liver injury is largely mediated by immunity. Therefore, it is plausible that ALT flares prior to HBsAg clearance is caused by the eradication of virus-infected hepatocytes by anti-viral CD8^+^ T-cell-mediated cytotoxicity and anti-viral humoral immunity [24]. It is also possible that ropeg has a direct effect on residual tumor cells or hepatocytes that may carry activated oncogenes due to the HBV infection by causing cell cycle inhibition and apoptosis [25, 26, 33]. The occurrence of ALT flares associated with the HBsAg clearance or decrease at 5/15 (33%) patients in our study suggests that the sequential combination regimen potentiates a significant response against the HBV-infected hepatocytes. The induction of anti-HBs antibody right after the ALT flares suggests that activation of an immune response is involved in the clearance of HBV-carrying hepatocytes.

The enticing phase 1 trial results warrant further study of this sequential regimen in post-resection HCC patients. An open-label, randomized, and multicenter phase 2 study is being planned to further evaluate the regimen of ropeg and anti-PD-1 treatment as adjuvant treatment for post-surgical recurrence of viral-related HCC. Patients with resected or ablated viral hepatitis-related HCC will be enrolled. Eligible patients will be randomized to receive six doses of ropeg every two weeks at the dose of 450 μg, followed by six doses of nivolumab at 0.75 mg/kg every two weeks versus control treatment. The anti-PD1 treatment increased to six doses was based on the study observation that two patients with ALT flares had HBsAg reduction without complete clearance under the sequential treatment regimen with three doses of nivolumab. HBsAg levels rebounded after the third dose of anti-PD-1 therapy in the two cases. Additional doses of anti-PD1 therapy might enhance the probability of HBsAg clearance.

In summary, our phase 1 results including long-term cancer recurrence and patient survival data support the notion that sequential combination regimen of ropeg and nivolumab is effective in suppressing the post-surgical, recurrence of HBV-related HCC. This is also consistent with the mechanisms of action of the treatment and our previous animal modeling data. A randomized phase 2 trial is being planned to further evaluate this regimen against the recurrence of viral-related HCC.

## Supplementary Information

Below is the link to the electronic supplementary material.

Supplementary file1 (DOCX 77 KB)
