# Supplementary material for: Sequential combination with ropeginterferon alfa-2b and anti-PD-1 treatment as adjuvant therapy in HBV-related HCC: a phase 1 dose escalation trial
Source: Hepatol Int. 2025 Apr 5;19(3):547–59. doi: 10.1007/s12072-025-10824-4 (PMC12137535; doi:10.1007/s12072-025-10824-4)
Supplement: Supplementary file 1 — Supplementary file1 (DOCX 77 KB) [file 12072_2025_10824_MOESM1_ESM.docx]

(b)

(a)

*

**Supplemental Figure 1. Pre-operative and post-operative AFP level for all eligible patients (a) and for those with baseline AFP ≥200 ng/ml (b). * p value < 0.05, t teat for the comparison between pre- and post-operations.**
